# Awareness of obstetric fistula and associated factors among women in reproductive age group attending public hospitals in southwest Ethiopia, 2021

**DOI:** 10.1186/s12978-021-01228-2

**Published:** 2021-09-15

**Authors:** Dessalegn Nigatu Rundasa, Tarekegn Fekede Wolde, Kenbon Bayisa Ayana, Abeya Fufa Worke

**Affiliations:** 1 Department of Nursing, College of Health Sciences, Mettu University, Metu, Ethiopia; 2 Department of Midwifery, College of Health Sciences, Mettu University, Metu, Ethiopia

**Keywords:** Awareness, Obstetric Fistula, Southwest Ethiopia

## Abstract

**Background:**

Obstetric fistula occurs in all developing countries but it is confined to the “fistula belt” across the northern half of Sub-Saharan Africa from Mauritania to Eritrea and in the developing countries of the Middle East and Asia. Ending obstetric fistula is critical to achieving Sustainable Development by 2030. So creating awareness on obstetrics fistula among women in the reproductive age group have a crucial role in reducing morbidity, mortality, and social stigma.

**Objective:**

To assess awareness on obstetric fistula and its associated factors among reproductive-age women attending governmental hospitals in southwest Ethiopia, 2021.

**Methods:**

An Institutional based cross-sectional study design was conducted among 413 women. The sample size was estimated by using a single population proportion formula. The collected data were coded and entered into EPI-data version 3.1 then exported to SPSS version 24 for descriptive and inferential analysis. Adjusted odds ratio (AOR) along with 95% confidence level was estimated to assess the strength of the association and variables with a p-value < 0.05 were considered to declare the statistical significance in the multivariable analysis in this study.

**Results:**

In this study, a total of 400 clients have participated in the study. The mean ages of participants were 30.26 (SD ± 8.525) years old. Education of women who cannot read and write are 85% less likely to have good awareness than women who are above the secondary level of education [AOR = 0.162; 95% CI (0.081–0.364)]. While Women who have primary education level are 83% less likely to have good awareness than women who are above the secondary level of education [AOR = 0.170; 95% CI (0.085–0.446)]. In addition, This study shows women who have not heard about obstetric complications are 54% less likely to have awareness of obstetric fistula than those who heard about obstetric complications [AOR = 0.458; 95% CI (0.368–0.643)].

**Conclusion:**

This study identifies that the educational level of women, history of pregnancy, distance to the nearby health institution, and awareness of obstetrics complications were the factors associated with awareness of reproductive age women on obstetrics fistula. Hence, increasing awareness on obstetric fistula plays a key role in averting this problem.

## Plain Language Summary

Globally, between 2 and 3 million women were affected by obstetric fistula. Moreover, obstetric fistula is common in developing nations, as around 60% of it was found in sub-Saharan the so-called fistula belt’ and middle east Asia. This devastating condition adversely affects both the Physical and Mental health of the women. There is little research conducted concerning the health burdens of the obstetric fistula in southwest Ethiopia. Therefore, this article provides a better understanding of the level of awareness on obstetric fistula and barrier (factors) that hinders the early detection and treatment of obstetric fistula.

In this study, a cross-sectional study design was employed and data were collected from 400 women of the reproductive age group who visited public hospitals in the Ilu Aba Bor Zone. Our study shows that educational status, has not heard about complications of obstetric fistula, being far distant from the health facility, and not being pregnant were found to be factors that hinder the early detection and management of obstetric fistula. In conclusion, the finding of this study reported a low magnitude of level of awareness on obstetric fistula as compared to other previous studies.

## Introduction

Vaginal Fistula is an abnormal communication between the vagina and adjacent tubular structures-usually bladder and rectum—leading to continuous leakage of urine or feces—through the vagina [1]. Globally, an estimated 50,000 to 100,000 women develop fistula annually and about 2 million women currently live with fistula, which is a burden in almost 60 countries. Moreover, obstetric fistula appears in all developing countries, including African and middle east Asia, particularly confined across the northern half of sub-Saharan Africa from Mauritania to Eritrea as called the fistula belt’ [2]. In Africa, Fistula is predominantly caused by prolonged and or obstructed labor; however, abdominal hysterectomy remains the most common cause of vaginal fistula in developed countries [3]. Currently, in Ethiopia, there are more than 110,000 women who have a vaginal fistula, of those less than 2000 women get treatment in the last 3 years [4].

Although Obstetric fistula is an indicator of the health system failing to provide accessible, timely, and appropriate intra-partum care [5]. Moreover, Obstetric fistula is devastating lifelong morbidity if left untreated, with severe medical, social, psychological, and economic consequences [4, 6–8]. The underlying factors contributing to Obstetrics fistula including no skilled birth attendants [9, 10], poor health-seeking behavior, poor referral system and transportation [11, 12] network [11, 13], age and physical maturity [12, 14–16], iatrogenic surgical damage[9], Educational status [10, 17, 18], sexual violence [16], poverty [11, 13], lack of awareness [5, 12, 15, 17] and not spacing between childbirths [9]. Despite these contributing factors, the main reason women prevent Fistula care was that lack of awareness of obstetric fistula prevention and treatment takes a lion share [6] and lack of awareness is a leading reason to seeking treatment [7]. Therefore, identify awareness and associated factors to have paramount importance to avert and or to reduce obstetric fistula among women of reproductive age categories. So that, measuring awareness and factors associated with obstetric fistula among women of reproductive age in southwest Ethiopia public hospitals is important as there are scares information in a study setting.

## Methods and materials

### Study area and setting

The study area was public hospitals in southwest Ethiopia. Which is located about 600 km away from the capital city, Addis Ababa, Ethiopia. It is bordered on the south by the south nation, nationality and Peoples’, on the southwest by the Gambela, on the west by kelem wollega zone, on the north by west wollega zone, on the northwest by East wollega zone, and on the east by Jimma zone. The zone has an estimated area of 15,135.33 KM2. Moreover, the area has five hospitals and thirty-nine health centers. We used the five hospitals.

### Study design and period

An institution-based cross-sectional study design was employed from December 30 to January 31, 2021.

### Source and study population

All women in the reproductive age group (i.e., literally age b/n 15–49 years) attending governmental hospitals in southwest Ethiopia visiting Gynecology out Patient Department (OPD) and Ante Natal Care (ANC).

### Study unit

Randomly selected reproductive age women and fulfill the inclusion criteria.

### Eligibility criteria

#### Inclusion criteria

All reproductive age group women those available at the time of the data collection period, volunteer and who are visiting the Gynecology Out Patient Department and Ante Natal Care.

#### Exclusion criteria

Women who refuse to participate in the study.

### Sample size determination

The sample size was estimated by using a single population proportion formula. By considering the prevalence level of obstetric fistula that can estimate maximum sample size (57.8%), marginal error (d) 0.05, with 95% confidence interval certainty and margin of error 0.05 was considered. Finally, by adjusting the 10% non-respondent rate the sample size turned out 413.

### Sampling procedure

Five public hospitals were selected. A simple random sampling technique was employed after study subjects were proportionally allocated to each hospital.

### Operational definitions

*Awareness* Conscious or informed about obstetric fistula cause, prevention, and presentation.

*Good Awareness* Participants who scored above the mean score whereas, Poor Awareness participants scored less than the mean.

### Data collection tool and technique

A structured interviewer-administered adopted questionnaire was used for data collection. The questionnaire was first prepared in English and then translated into the local naïve language ‘Afan Oromo’ and re-translated back into English to maintain consistency by a fluent bilingual expert. Data were collected from each study subject by BSc holder midwifery in face-to-face interview from December 30 to January 30, 2021 at each hospital.

### Data quality control

A two days training was given for both data collectors and supervisors on data collection procedure, accuracy, and completeness of the data. After the training, a pretest was carried out on the 5% of the sample was done in a similar population that was in another hospital, to ensure the quality and validity of the data. Finally, the supervisor checked for completeness on regular basis.

### Data processing and analysis

Data were checked for completeness, clarity, and consistency. The data were entered into EPI- data version 3.1 and exported to SPSS version 24 statistical software for analysis. Descriptive statistics were employed. Logistic regression was used to see the association between dependent and independent variables. Statistical significance was declared at P-value less than 0.05 at 95% CI as the cut of point. Odds Ratio at 95% CI was used to identify the presence and strength of association.

### Ethical consideration

To conduct the study an ethical approval was obtained from Institutional Review Board of College of Health Sciences, Mettu University, Ethiopia. Official letter of cooperation was written to south west health bureau from department of public health. Written, informed and signed consent was taken from respondents of age 18 and above after telling, the confidentiality was kept and the role of their participation is for research purpose.

## Results

### Socio-demographic characteristic

Out of a total of 413, 400 respondents were participated, with a response rate of 96.85%. The mean age of participants was 30.26 (SD ± 8.525) years old. More than half (55%) of participants were Orthodox Christian in religion. The majority of participants were Oromo in ethnicity (Table1).

**Table 1:** Socio demographic characteristics of participants among women in reproductive age group attending public hospitals in southwest Ethiopia, 2021 (n = 400)

Variable	Frequency	Percent
Age		
< Mean (30.26 years)	208	52
> Mean (30.26 years)	192	48
Religion		
Orthodox Christian	220	55.0
Muslim	133	33.3
Catholic	12	3
Protestant	35	8.8
Ethnicity		
Oromo	256	64
Amhara	98	24.5
Gurage	29	7.3
Others	17	4.3
Marital status		
Single	100	25.0
Married	254	63.5
Divorced	20	5.0
Separated	11	2.8
Widowed	15	3.8
Marriage age		
15–19	112	28.0
20–24	142	35.5
25–29	26	6.5
30–34	5	1.3
35–39	1	0.3
Educational level		
No formal education	89	22.2
Primary education	104	26.0
Secondary education	112	28.0
Above secondary	95	23.8
Occupation		
House wife	193	48.3
Student	65	16.3
Farmer	9	2.3
Merchant	42	10.5
Government employee	40	10.0
Private employee	51	12.8
Monthly Income (Ethiopian Birr)		
≤ 1000	167	41.8
1001–2000	129	32.3
2001–3000	54	13.5
3001–4000	25	6.3
4001–5000	25	6.3

### Obstetric characteristic

Out of a total, 205 (51.1%) participants do not hear about obstetrical complications. Almost half of the participants (49.8%) use modern family planning methods. Women who have less than three pregnancies are 170 (42.5%) (Table 2).

**Table 2:** Obstetric characteristic of participants among women in reproductive age group attending public hospitals in southwest Ethiopia, 2021

Variable	Frequency	Percent
Heard about obstetric complication		
Yes	195	48.9
No	205	51.1
History of induced abortion (n = 285)		
Yes	62	21.8
No	223	78.2
History of birth complication (n = 285)		
Yes	122	42.8
No	163	57.2
Contraceptive use		
Yes	197	49.3
No	203	50.8
Type of contraceptive used (n = 197)		
Injectable	106	53.8
Pills	41	20.8
Implants	35	17.8
IUCD	12	6.1
Condom	3	1.5
Have you ever an pregnant		
Yes	253	63.3
No	147	36.8
Age at 1st pregnancy		
15–19	112	39.2
20–24	142	49.7
25–29	26	9.1
30–34	5	1.7
35–39	1	0.3
Number of parity		
1–3	170	42.5
4–6	90	22.5
7–9	21	5.3
10–12	4	1.0

### Awareness on obstetric fistula

Women who have ever heard of obstetric fistula are 53% and out of these, 34.6% of women get information from media about obstetric fistula. Moreover, 38.1% of them reported the inability to control urine as a symptom of obstetric fistula (Table 3).

**Table 3:** Participants characteristics about Awareness on Obstetric fistula among women in reproductive age group attending public hospitals in southwest Ethiopia, 2021

Variable	Frequency	Percent
Ever heard about obstetric fistula		
Yes	212	53.0
No	188	47.0
Symptoms of obstetric fistula you heard		
Unable to control urine	82	38.1
Unable to control feces	4	1.9
Unable to control urine and feces	58	27.0
Bleeding and pain during sex	23	10.7
Abnormal Vaginal discharge	23	10.7
Irritation during urination	25	11.6
Source of information about obstetric Fistula		
Health facility	56	26.2
Family and friends	47	22.0
School	15	7.0
Media	74	34.6
Victim of fistula	22	10.3

**Fig. 1:**
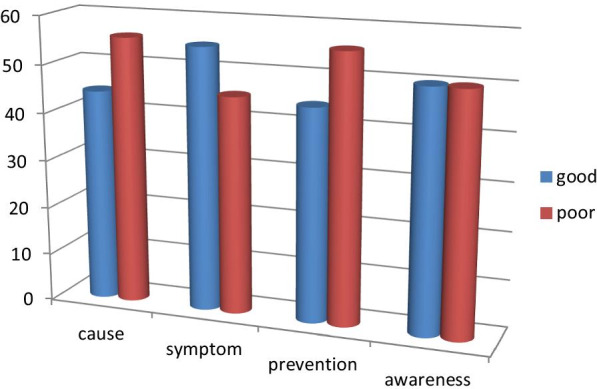
The status of Women's awareness on obstetric fistula at public hospitals in southwest Ethiopia, 2021

Women who have a good awareness of causes, symptoms, and prevention are 177 (44.3%), 219 (54.8%), and 177 (44.3%) respectively. General Obstetric fistula awareness of women in visiting hospitals in southwest Ethiopia is 50%, which is poor awareness on obstetric fistula. (Fig. 1).

### Factors associated with awareness status on obstetric fistula

In bivariate analysis, the result shows educational status of women, distance to the health center, modern contraceptive use, hearing about delivery complications, and history of pregnancy were found to have significant associations.

In multivariate analysis, the result showed a significant association between history of pregnancy and the odds of having good awareness. Those reproductive age group who had a history of pregnancy were 68% less likely to have a good awareness of obstetrics fistula prevention than their counterparts [AOR = 0.399; 95% CI (0.241–0.661)]. Distance to the nearby health institution (hospital), those who lived above half an hour foot distance were 65% less likely to have good awareness than those living near to health facility [AOR = 0.355; 95% CI (0.212–0.594)]. Education of women who cannot read and write are 85% less likely to have good awareness than women who are above the secondary level of education [AOR = 0.162; 95% CI (0.081–0.364)]. Women who have primary education level are 83% less likely to have good awareness than women who are above the secondary level of education [AOR = 0.170; 95% CI (0.085–0.446)] and women who have secondary education level are 70% less likely to have good awareness than women who are above the secondary level of education [AOR = 0.299; 95% CI (0.153–0.584)] In addition, my study shows women’s who have not heard about obstetric complication are 54% less likely to have awareness on obstetric fistula than those who heard about obstetric complication [AOR = 0.458; 95% CI (0.368–0.643)] (Table 4).

**Table 4:** Multivariate analysis showing the impact of selected associated factors of obstetric fistula among reproductive age women in in public hospitals in southwest Ethiopia, 2021

Variable	Awareness on OF				
	Good	Poor	COR (95% CI)	AOR (95% CI)	p value
Women education					
Can’t read and write	**30 (33.7%)**	**59 (66.3%)**	**0.119 (0.060–0.234)**	**0.162 (0.081–0.364)**	0.000**
Primary	**41 (39.4%)**	**63 (60.6%)**	**0.152 (0.080–0.290)**	**0.170 (0.085–0.446)**	0.001*
Secondary	**52 (46.4%)**	**60 (53.6%)**	**0.203 (0.108–0.382**	**0.299 (0.153–0.584)**	0.000**
Above secondary	77 (81.1%)	18 (18.9%)	1		
HDC					
No	**81 (39.7%)**	**123 (60.3%)**	**0.430 (0.288–0.642)**	**0.458 (0.368–0.678)**	0.000**
Yes	118 (60.5%)	77 (39.5%)	1	1	
Distance to HF on foot by minutes					
> 30	**36 (26.5%)**	**100 (73.5%)**	**0.220 (0.139–0.346)**	**0.355 (0.212–0.594)**	0.000**
≤ 30	164 (62.3%)	100 (37.7%)	1	1	
Contraceptive use					
No	91 (44.8%)	112 (55.2%)	0.656 (0.442–0.973)	1.101 (0.601–2.013)	0.712
Yes	109 (55.3%)	88 (44.7%)	1	1	
Ever pregnancy					
No	**46 (31.3%)**	**101 (68.7%)**	**0.293 (0.190–0.450)**	**0.399 (0.241–0.661)**	0.002*
Yes	154 (60.9%)	99 (39.1%)	1	1	

*OF* obstetric-fistula, *HF* health facility, *HDC* heard delivery complication

**Significant association (p < 0.01)

## Discussion

In this study, half of respondents 50% at 95% confidence interval (34.62–45.37) have had good awareness about obstetric fistula. Despite the problems associated with obstetric fistula, many women are not aware of the obstetric fistula. It may be difficult to control a disease that people are not aware of. This finding may also imply that these women were not aware of how to prevent obstetric fistula. This finding was lower when compared with the study done in Delanta district which is 55.4% [19, 20], the difference might be due to sampling size and study participants. Our finding was consistent when compared to a study done in Ghana which was 47.6% [15].

This study showed that 212 (53%) reproductive age group women have heard obstetric fistula this result is higher than EDHS, 2016 of which 2 in 5 women interviewed in the survey had heard of obstetric fistula (39%) [2].This difference might be due to EDHS takes sample from different regions of Ethiopia which have different geographical location, social and cultural factors and my study took only participants who visit hospitals.

My study shows there is significant association between women awareness and their educational level, women’s who cannot read and write are 84% less likely to have good awareness than women who are above secondary level of education. Women’s who have primary education level are 83% less likely to have good awareness than women who are above secondary level of education and women’s who have secondary education level are 70% less likely to have good awareness than women who are above secondary level of education. This shows that when education level is improved the awareness comes more improved. The finding also supported by study conducted in Cameroonian women which shows 53% of women who had no previous knowledge on obstetric fistula were generally the illiterate [3]. Another study, which supports my study, is survey on obstetric fistula awareness which was done in Northern Ghana, how that Level of education of the participants had a significant influence on.

Awareness the level of fistula with about 50.2% those with higher education aware of as.

Compared to 49.8% who had no knowledge about fistula the result of this study showed that there were a significant association tween women’s awareness and distance to the nearby health center. Women living in areas where hospital or health institution is found at a distance more than half an hour (on foot) from the home they are living, their awareness is 65% less likely to good than those living in areas which are found at a distance less than or equal to half an hour. This can supported by studies in Ethiopia that identifies distance to the health care facility was linked with poor knowledge related to fistula and poor health seeking behavior which increases the likelihood of more fistula cases [4]. A study on awareness to health knowledge in Western China showed that health knowledge declined as the distance from the nearby health institution increases [5].This might cause of, as the health facility comes near women’s are to visit the facility cause they do not need transportation.

In my study women’s who have not heard about obstetric complication are 54% less likely to have awareness on obstetric fistula than those who heard about obstetric complication. This finding is supported by study done in Amhara region which shows the presence of awareness about complications of delivery other than obstetrics fistula was significantly associated with sufficient knowledge on obstetrics fistula. This might cause of the issue that obstetrics fistula can also informed together with dangers of pregnancy since it is among the child birth complications.

This study showed that good awareness on obstetric fistula tended to more common among women with previous pregnancy history than those without. Those who had history of pregnancy were 60% less likely to have good awareness. This finding is supported by study done in Delanta district which shows, women with no pregnancy history were 87% times less likely to have sufficient knowledge. The finding is also consistent with other studies which showed non-exposure to pregnancy prevents women's knowledge on obstetrics fistula by 80% [6].

## Conclusion

The finding indicates that 50% of reproductive age women have poor awareness about obstetric fistula, and Educational level of women, history of pregnancy, distance from health facility and awareness on obstetrics complications were the factors associated with awareness of reproductive age women on obstetrics fistula.

*Limitations* As it is a cross sectional study; it cannot show cause-effect relationship between factors and outcome variable and the same to other behavioral studies, respondents might not reply openly to sensitive and private questions.

## Data Availability

All data supporting our findings will be shared upon request.

## Abbreviations

CS: Caesarean section
EDHS: Ethiopian demographic and health survey
DH: Darimu hospital
FGD: Focused group discussions
IDI: In-depth interviews
IRB: Institutional Review Board
OF: Obstetric fistula
MKH: Mettu Karl Hospital
RVF: Recto vaginal fistula
SPSS: Statically package for social sciences
TBA: Traditional birth attendants
UNFPA: United Nation Fund for Population Agency
USAID: United States Agency for International Developments
VVF: Vesico-vaginal fistula
